# Type 2 diabetes mellitus increases the mortality risk after acute coronary syndrome treated with coronary artery bypass surgery

**DOI:** 10.1186/s12933-020-01069-6

**Published:** 2020-06-13

**Authors:** Eilon Ram, Leonid Sternik, Robert Klempfner, Zaza Iakobishvili, Enrique Z. Fisman, Alexander Tenenbaum, Elchanan Zuroff, Yael Peled, Ehud Raanani

**Affiliations:** 1 Department of Cardiac Surgery, Leviev Cardiothoracic and Vascular Center, Sheba Medical Center, 52621 Tel Hashomer, Israel; 2 Department of Cardiology, Leviev Cardiothoracic and Vascular Center, Sheba Medical Center, 52621 Tel Hashomer, Israel; 3 Sackler School of Medicine, Tel Aviv University, Tel Aviv, Israel; 4 Clalit Health Services, Tel Aviv, Israel

**Keywords:** Diabetes mellitus, Coronary artery bypass grafting, Revascularization, Insulin

## Abstract

**Background:**

Type 2 diabetes mellitus (DM) is a risk factor for cardiovascular diseases and is common among patients undergoing coronary artery bypass grafting (CABG) surgery. The main objective of our study was to investigate the impact of DM type 2, and its treatment subgroups, on short- and long-term mortality in patients with acute coronary syndrome (ACS) who undergo CABG.

**Methods:**

The study included 1307 patients enrolled from the biennial Acute Coronary Syndrome Israeli Survey between 2000 and 2016, who were hospitalized for ACS and underwent CABG. Of them, 527 (40%) patients were with and 780 (60%) were without DM.

**Results:**

Compared with the non-diabetic group, the diabetic group of patients comprised more women and had more comorbidities such as hypertension, dyslipidemia, renal impairment, peripheral vascular disease and prior ischemic heart disease. Overall 30-day mortality rate was similar between DM and non-DM patients (4.2% vs. 4%, p = 0.976). Ten-year mortality rate was higher in DM compared with non-diabetic patients (26.6% vs. 17.7%, log-rank p < 0.001), and higher in the subgroup of insulin-treated patients compared to non-insulin treated patients (31.5% vs. 25.6%, log-rank p = 0.019). Multivariable analysis showed that DM increased the mortality hazard by 1.61-fold, and insulin treatment among the diabetic patients increased the mortality hazard by 1.57-fold.

**Conclusions:**

While type 2 DM did not influence the in-hospital mortality hazard, we showed that the presence of DM among patients with ACS referred to CABG, is a powerful risk factor for long-term mortality, especially when insulin was included in the diabetic treatment strategy.

## Background

Currently, overall cardiovascular disease affects approximately 32.2% of all type 2 diabetes mellitus (DM) patients worldwide, while cardiovascular disease is a major cause of mortality among people with type 2 DM, accounting for approximately half of all deaths [1]. Diabetic patients presenting with acute coronary syndrome (ACS) have poor prognoses due to the diffuse and rapidly progressive forms of atherosclerosis and multiple comorbidities [2].

Previous studies have demonstrated increased short- and long-term mortality in diabetic patients undergoing coronary artery bypass grafting (CABG) or even isolated valve surgery [3] compared with non-diabetic patients [4, 5], and more recent reports have shown a significant reduction in mortality among patients with diabetes [6]. However, none of these studies were performed on ACS patients.

The main objective of our study was to investigate the impact of DM type 2, and its treatment subgroups, on short- and long-term mortality in patients with ACS who undergo CABG.

## Methods

### Study design

The ACS Israeli Survey (ACSIS) is a voluntary biennial prospective national registry of all patients with ACS hospitalized in the 25 coronary care units and cardiology departments in all the public health hospitals in Israel over a 2-month period (from March to April) [7].

ACSIS is managed by the Working Group on Acute Cardiovascular Care of the Israel Heart Society, in participation with the Israeli Center for Cardiovascular Research. Demographic, historical, and clinical data from all patients were recorded on pre-specified forms. Patient management was at the discretion of the attending physicians. Admission and discharge diagnoses were recorded as determined by the attending physicians based on clinical, electrocardiographic, and biochemical criteria.

### Study population

All patients in each medical center signed an informed consent form prior to participating in the ACSIS registry, and each center received approval from its institutional review board [8]. Between 2000 and 2016 (which included 8 consecutive registries), 1307 patients were hospitalized with ACS and underwent CABG and were included in the ACSIS registry. Of them, 527 (40%) patients had DM and 780 (60%) were without DM.

### Clinical outcomes

Clinical outcomes included 30-day major adverse cardiovascular events (MACE: which included death, MI, and stroke), in-hospital complications, and long-term all-cause mortality.

### Data collection and follow-up

All data from the 25 participating hospitals were collected and pooled into a designated database. All centers used standardized definitions for data collection, including demographic parameters, medical history, chronic and peri-procedural medical treatment, echocardiography measurements, procedure information and outcome measures. All patients were prospectively followed up for clinical events at 30 days and for late mortality. Mortality data were ascertained from the Israeli Ministry of Interior Population Registry through January 2018.

### Statistical analysis

Data are presented as mean ± standard deviation for normal, or median for abnormal distribution. Continuous variables were tested with the Kolmogorov–Smirnov test for normal distribution. Categorical variables are given as frequencies and percentages. A Chi square test was used for comparison of categorical variables between the groups, a Student t-test was performed for comparison of normally distributed continuous variables, and a Mann–Whitney U test for non-normal distribution.

Multivariable logistic regression analysis was used to identify factors associated with 30-day mortality. All statistically different variables (p < 0.1) in Table 1 and pre-specified variables were entered into the model. Long-term survival analysis was carried out using the Kaplan–Meier method, and comparison by the groups was tested using the log-rank test. Cox proportional hazard model was constructed to assess the association between DM and 10-year mortality adjusted to the following covariates: age, gender, hypertension, dyslipidemia, smoking, body mass index, renal impairment, prior myocardial infarction (MI), prior stroke, and congestive heart failure. Variables that were significant by the univariable analysis (p < 0.1) were included in the model. Results are presented as hazard ratio (HR), 95% confidence interval (CI) and p-value.

**Table 1:** Patient characteristics

	Diabetes mellitus No. of patients (527) (%)	Non-diabetic No. of patients (780) (%)	p value
Age, years (mean ± SD)	65 ± 10	64 ± 12	0.096
Sex (male)	399 (76)	645 (83)	0.003
Hypertension	375 (71)	411 (53)	< 0.001
Current smokers	158 (30)	286 (37)	0.020
Dyslipidemia	399 (76)	486 (63)	< 0.001
COPD	21 (5)	27 (5)	0.641
Family history of CAD	118 (24)	190 (25)	0.632
BMI (kg/m^2^) (mean ± SD)	28.4 ± 8	28 ± 16.4	0.701
Prior MI	154 (29)	213 (27)	0.445
Prior PCI	157 (30)	156 (20)	< 0.001
Renal impairment	61 (12)	44 (6)	< 0.001
Peripheral vascular disease	63 (12)	60 (8)	0.013
CVA/TIA	54 (10)	59 (8)	0.108
Congestive heart failure	55 (10)	33 (4)	< 0.001
On-site cardiac surgery unit	274 (52)	417 (53)	0.642
*Prior medications*			
Insulin	89 (20)	0 (0)	< 0.001
Oral antihyperglycemic agents	320 (69)	0 (0)	< 0.001
Aspirin	297 (65)	294 (44)	< 0.001
Clopidogrel	43 (9)	37 (6)	0.015
ACE-I	148 (48)	97 (23)	< 0.001
ARBs	42 (13)	32 (8)	0.015
Beta blockers	197 (43)	217 (33)	0.001
Statins	266 (59)	262 (40)	< 0.001
Calcium channel blockers	122 (27)	113 (17)	< 0.001
Nitrates	67 (15)	78 (12)	0.169
Aldosterone receptor antagonist	2 (1)	2 (1)	1.000
Diuretics	75 (20)	70 (13)	0.004

*SD* standard deviation, *COPD* chronic obstruction pulmonary disease, *CAD* coronary artery disease, *BMI* body mass index, *MI* myocardial infarction, *PCI* percutaneous coronary intervention, *CVA* cerebrovascular accident, *TIA* transient ischemic attack, *ACE-I* angiotensin converting enzyme inhibitors, *ARB* angiotensin II receptor blockers

Statistical significance was assumed when the null hypothesis could be rejected at p < 0.05. All p-values reflect results of two-sided tests. Statistical analyses were conducted using R (version 3.4.1).

## Results

### Baseline characteristics

In our study cohort there were 780 non-diabetic patients, and 527 patients with DM type 2. Of them, 273 were treated with oral antihyperglycemic medications, 89 with insulin (with or without oral antihyperglycemic medications), and 165 with diet only. Presentation of the ACS was ST-segment elevation MI in 35%, non-ST-segment elevation MI in 45% and unstable angina pectoris in 20% (with no difference between DM and non-DM patients, p = 0.109). Compared with the non-diabetic group, the diabetic group of patients were more frequently women and had more comorbidities such as hypertension, dyslipidemia, renal impairment, peripheral vascular disease and prior ischemic heart disease (Tables 1 and 2). In addition to the antihyperglycemic medication, patients with DM were treated more frequently with platelet anti-aggregation therapy, angiotensin converting enzyme inhibitors or angiotensin II receptor blockers, calcium channel blockers, statins and diuretics (Table 1).

**Table 2:** Acute coronary syndrome presentation

	Diabetes mellitus No. of patients (527) (%)	Non-diabetic No. of patients (780) (%)	p-value
ACS diagnosis			0.109
NSTEMI	253 (48)	333 (43)	
STEMI	169 (32)	291 (37)	
UAP	105 (20)	156 (20)	
Left ventricle ejection fraction			< 0.001
Normal (> 50%)	140 (31)	281 (44)	
Mild (40–50%)	158 (36)	177 (27)	
Moderate (30–40%)	97 (22)	133 (21)	
Severe (< 30%)	48 (11)	52 (8)	
Number of CAD			< 0.001
1 Vessel	18 (5)	43 (8)	
2 Vessels	77 (20)	150 (29)	
3 Vessels	286 (75)	323 (63)	
*Vital signs on admission*			
Heart rate (bpm) (mean ± SD)	87 ± 21	81 ± 20	< 0.001
Systolic blood pressure (mmHg) (mean ± SD)	144 ± 29	143 ± 29	0.569
Diastolic blood pressure (mmHg) (mean ± SD)	80 ± 16	83 ± 17	0.013
Normal sinus rhythm	417 (90)	615 (91)	0.795
Atrial fibrillation/SVT	24 (6)	22 (4)	0.122
VT/VF	1 (0.3)	5 (0.9)	0.447
2-3-degree AV-Block	3 (1.2)	3 (0.8)	0.947

*ACS* acute coronary syndrome, *NSTEMI* non-ST-segment elevation myocardial infarction, *STEMI* ST-segment elevation myocardial infarction, *UAP* unstable angina pectoris, *CAD* coronary artery disease, *SD* standard deviation, *SVT* supraventricular tachycardia, *VT* ventricular tachycardia, *VF* ventricular fibrillation, *AV* atrioventricular

### Early outcomes

Overall 30-day mortality rate was similar between the DM and non-DM patients (4.2% vs. 4%, p = 0.976), and between the subgroups of insulin-treated DM and non-insulin-treated DM (5.7% vs. 3.9%, p = 0.633). Other 30-day major events were similar between the DM and non-DM patients, such as stroke (0% vs. 0.3%, p = 0.658), recurrent MI (1.5% vs. 1.7%, p = 1.000) and MACE (p = 0.264). Major events were also similar between the non-insulin dependent and insulin-dependent DM patients: stroke (0% vs. 0%, p = 1.000), recurrent MI (0% vs. 1.8%, p = 0.415) and MACE (p = 0.615). These results were similarly consistent in the subgroups of the different ACS presentations and were reported as counts and crude event rates (Table 3).

**Table 3:** Early (30-day) crude counts and event rate by the acute coronary syndrome presentation

	STEMI			NSTEMI			UAP		
	DM N = 169	Non-DM N = 291	p-value	DM N = 253	Non-DM N = 333	p-value	DM N = 105	Non-DM N = 156	p-value
Death	10 (6)	15 (5)	0.900	9 (4)	14 (4)	0.850	3 (3)	2 (1)	0.653
Recurrent MI	4 (2)	7 (2)	1.000	4 (2)	5 (2)	1.000	0 (0)	1 (1)	1.000
CVA/TIA	0 (0)	1 (0)	1.000	0 (0)	1 (0)	1.000	0 (0)	1 (1)	1.000
MACE*	7 (4)	17 (6)	0.412	7 (3)	12 (4)	0.496	2 (2)	1 (1)	0.787

*STEMI* ST-segment elevation myocardial infarction, *NSTEMI* non-ST-segment elevation myocardial infarction, *UAP* unstable angina pectoris, *DM* diabetes mellitus, *MI* myocardial infarction, *CVA* cerebrovascular accident, *TIA* transient ischemic attack, *MACE* major adverse cerebrovascular event

* MACE includes 30-day mortality, myocardial infarction, and stroke

**Fig. 1:**
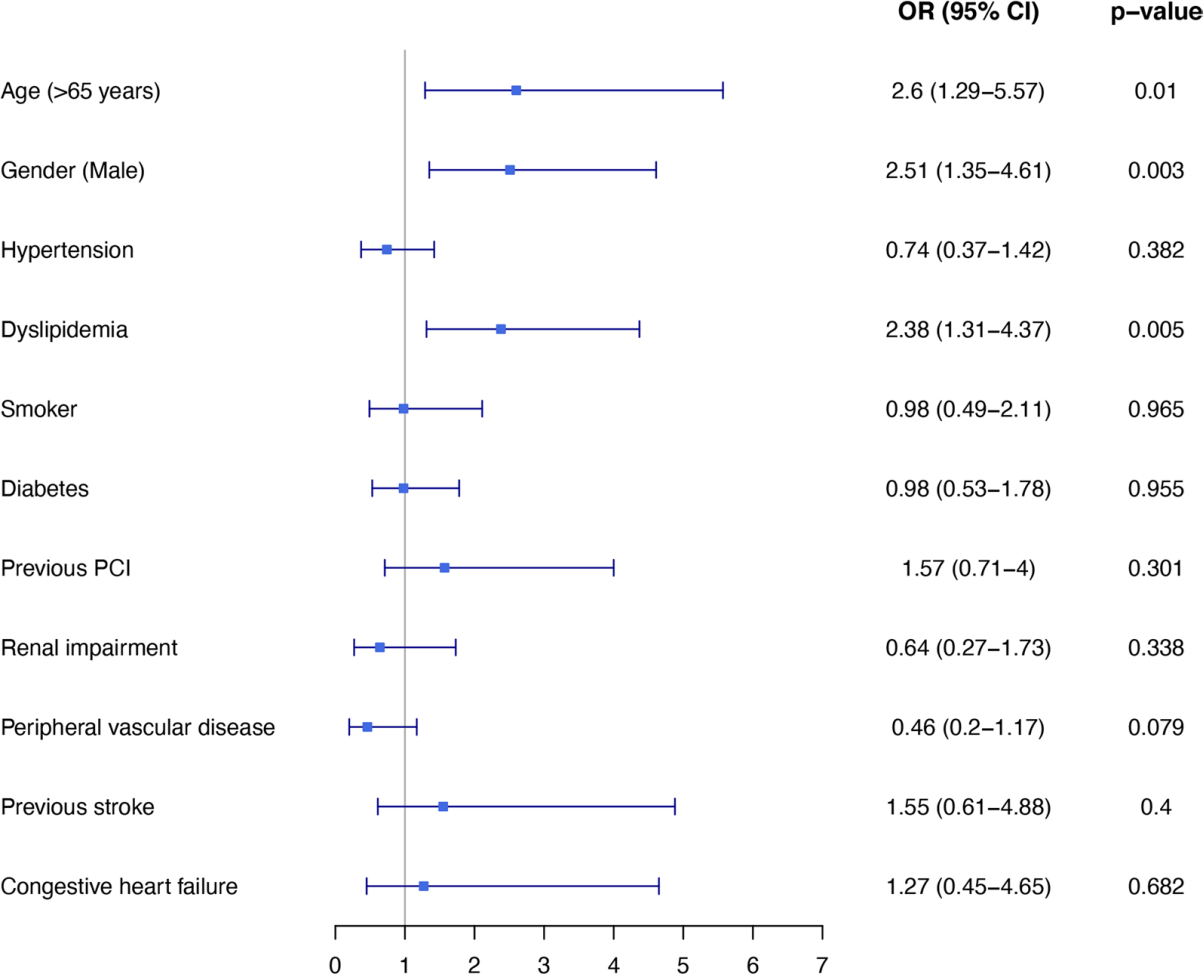
Forest plot: Predictors for 30-day mortality using logistic regression analysis. Odds ratio for 30-day mortality with 95% confidence interval. *OR* odds ratio, *CI* confidence interval, *PCI* percutaneous coronary intervention

Multivariable logistic regression analysis demonstrated that DM was not a predictor for death at 30-days after CABG (OR 0.98 95% CI 0.53–1.78, p = 0.955). The only significant variables that were associated with 30-day mortality rate were older age, male gender and dyslipidemia (Fig. 1).

### Long-term mortality rate

**Fig. 2:**
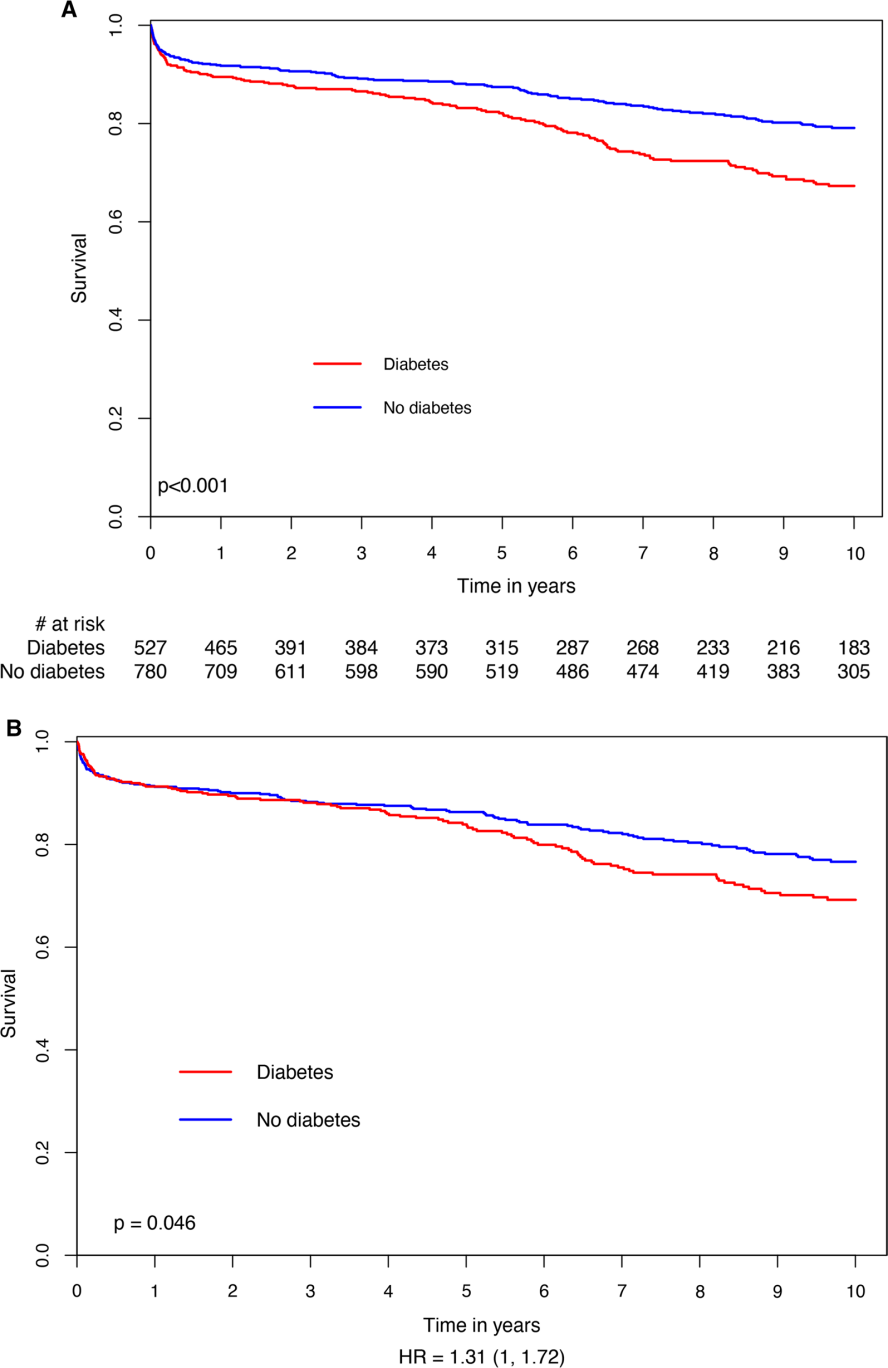
**a** Unadjusted 10-year survival curves by the presence of diabetes mellitus. **b** Hazard plot for survival at 10 years by the presence of diabetes mellitus, with propensity score adjustment. The covariates included in the model were: age, gender, hypertension, dyslipidemia, smoking, body mass index, renal impairment, prior MI, prior stroke and congestive heart failure. *HR*  hazard ratio, *MI* myocardial infarction

Kaplan–Meier survival analysis showed that mortality rates at 10 years of follow-up among patients with DM were significantly higher (26.6%) compared with those without DM who had ACS treated by CABG (17.7%; log-rank p-value < 0.001 for the overall difference during follow-up [Fig. 2a]). Consistent with the univariable findings, adjusted analysis, including a propensity score for the presence of DM, using a propensity score for confounders, also demonstrated a significantly higher risk for 10-year mortality among patients with, compared to those without DM (Fig. 2b). Additional independent predictors for long-term mortality among all study patients included: age > 65 years, male gender, hypertension, dyslipidemia and renal impairment (Table 4).

**Table 4:** Multivariable Cox regression analysis—predictors for 10-year all-cause mortality

	HR	95% CI	p-value
Diabetes mellitus	1.34	1.03–1.76	0.032
Age > 65 years	2.22	1.61–3.08	< 0.001
Sex (male)	0.68	0.51–0.92	0.014
Hypertension	1.57	1.14–2.17	0.006
Dyslipidemia	0.62	0.47–0.82	0.001
Current smoker	0.98	0.70–1.36	0.910
BMI	0.97	0.94–1.00	0.054
Renal impairment	1.59	1.07–2.35	0.020
Prior MI	1.22	0.91–1.63	0.190
Prior CVA/TIA	0.91	0.60–1.38	0.657
History of CHF	1.15	0.73–s1.81	0.543

*HR* hazard ratio, *CI* confidence interval, *BMI* body mass index, *MI* myocardial infarction, *CVA* cerebrovascular accident, *TIA* transient ischemic attack, *CHF* congestive heart failure

**Fig. 3:**
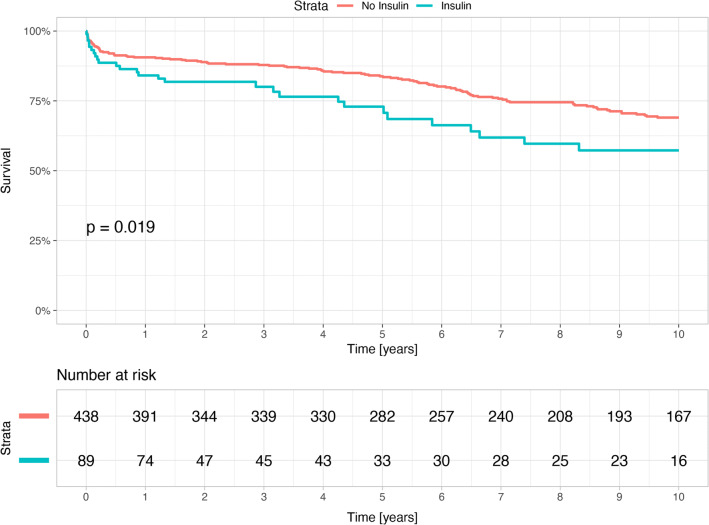
Kaplan Meier curves for survival in the diabetes mellitus group by insulin treatment

**Fig. 4:**
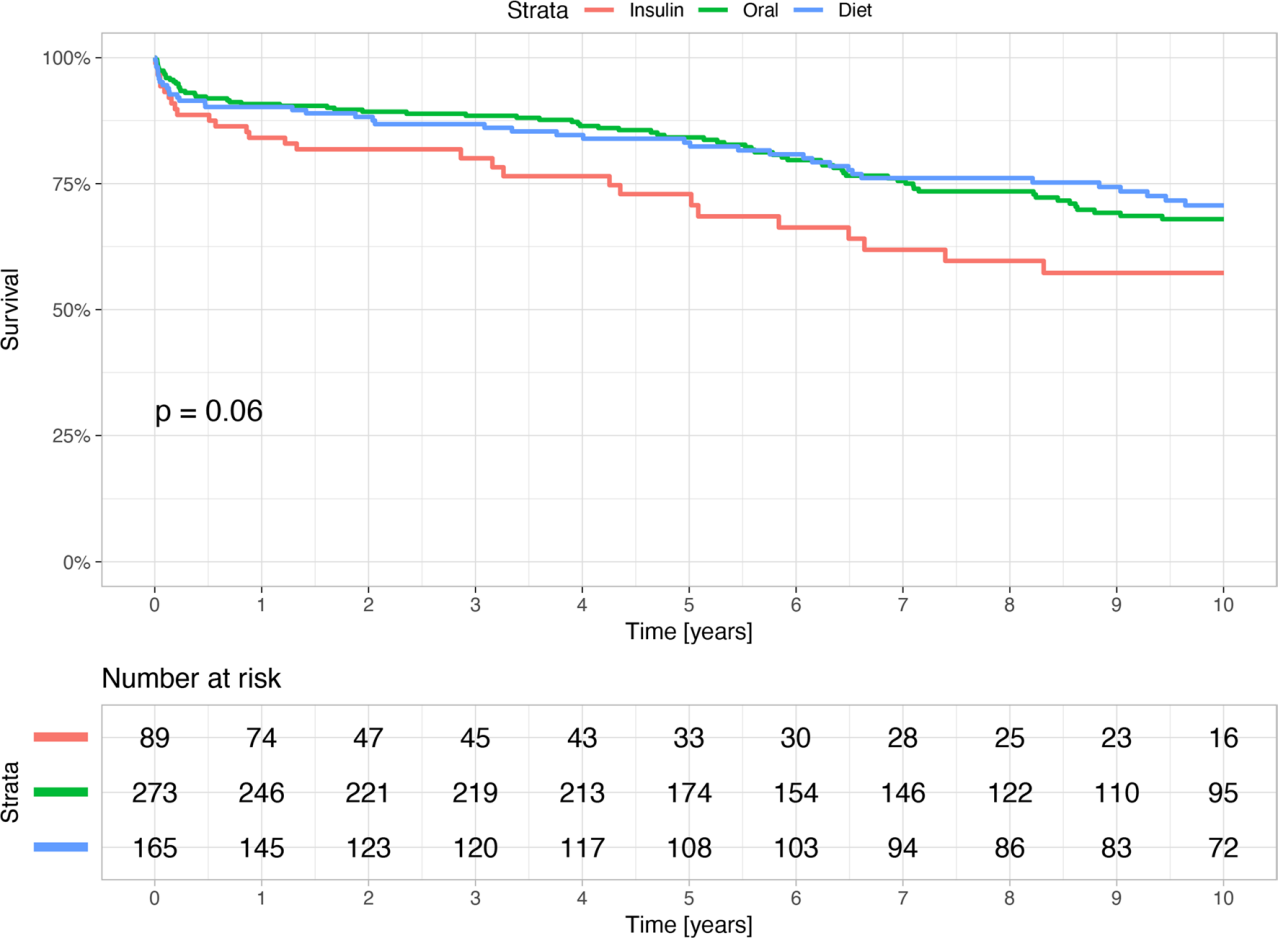
Kaplan Meier curves for survival in the diabetes mellitus group by the three treatment categories

Furthermore, long-term mortality was higher in the subgroup of insulin-treated patients compared to the subgroup of non-insulin treated patients with 10-year mortality rates of 31.5% vs. 25.6% (p = 0.019, Fig. 3). Interestingly, there were no significant differences in long-term mortality in DM patients treated with oral antihyperglycemic drugs or with diet only (Fig. 4).

## Discussion

Our observational real-world study investigated the impact of type 2 DM on early- and long-term mortality in patients after ACS treated by CABG. First, we found that diabetic and non-diabetic patients, and insulin-dependent and non-insulin-dependent DM patients, had similar in-hospital outcomes. Second, our principal finding was that the long-term mortality rate of diabetic patients was higher than that of non-diabetic patients, and mortality was even higher when the diabetic treatment strategy included insulin.

### Long-term mortality

While not performed on an ACS population, prior studies of DM versus non-DM in patients who underwent CABG have shown inconsistent results regarding long-term mortality. As in our results, Marui et al. reported an increase in 3- and 5-year mortality rates (11% vs. 9.7% and 19.6% vs. 16.2%, respectively) [9], and Koshizaka et al. reported significant differences in 5-year mortality rates (15.5% vs. 8.5%) in diabetic compared to non-diabetic patients [10]. Wit et al. reported significantly higher 3-year mortality rates in patients with insulin-treated compared with non-insulin-treated DM and non-diabetic patients (16.7% vs. 8.7% vs. 6.3%) [11]. A previous report of our group among all CABG patients showed a 5-year mortality rate of 15.3% among diabetic patients and a 9.3% rate among non-diabetic patients [5]. In contrast, Onuma et al. reported slightly increasing mortality rates in diabetic compared with non-diabetic patients 5 years after CABG: 8.6% vs. 7.1% [12], and Kappetein et al. reported non-significant differences between diabetic and non-diabetic patients at 5 years: 12.9% vs. 10.9% [13]. While in the general population DM is associated with excess mortality, compared with the general population without DM, with a hazard ratio of 1.15 at 5 years [14], we reported a greater impact of DM on patients who underwent CABG (HR of 1.44 at 5 years and 1.61 at 10 years). We assume that this higher impact of DM in our cohort, compared to the natural history of DM in the general population, is due to accelerated coronary artery disease (CAD) [15].

### Operative mortality

We report here that in-hospital mortality among diabetic and non-diabetic patients after ACS was 4.2% and 4%, respectively. Furthermore, we have shown that DM was not associated with short-term cardiovascular events after CABG. Although patients with stable CAD were also studied, other comparisons between DM and non-DM patients who underwent CABG showed no difference in early outcomes. Abizaid et al. reported similar in-hospital mortality rates between diabetic and non-diabetic patients (2.1% vs. 1.2%) [16]. Marui et al. found no differences in 30-day mortality (0.9% vs. 1.2%) [9], as did Carson et al. (3.7% vs. 2.7%) [4]. Likewise, Li et al. reported similar post-CABG mortality rates for diabetic and non-diabetic patients (2% vs. 1.9%) [17]. Although diabetic patients in our series were older and had more comorbidities, differences in early mortality rates did not reach statistical significance.

### Cardiovascular risk factors and ACS

The DM group in our study included patients who were treated either with insulin, oral antihyperglycemic drugs or with diet. Interestingly, not only overt diabetes, but also genetic predisposition to type 2 DM was significantly associated with a greater severity of coronary atheromatous burden in patients with ACS, independently of traditional risk factors [18]. There were substantial differences between the DM and non-DM groups in our cohort, with many of the unfavorable clinical characteristics (gender, comorbidities, and lower left ventricle ejection fraction) being more common in the DM group. We attempted to overcome some of the clinical differences through statistical adjustment of important variables.

We reported that the prevalence of women was higher in the DM group. The fact that diabetic women presented with more comorbidities is in keeping with recent findings showing that, in both percutaneous and surgical revascularization, women presented with worse outcomes at 1 year; albeit there were no gender differences at 5 years of follow-up [19]. Previous studies have shown that women were shown to have significantly smaller epicardial coronary arteries than men, even after adjustment for age, body habitus, and left ventricular mass [20, 21]. Consequently, diverse and gender-specific pathophysiological processes may contribute to different outcomes seen in women as compared to men.

While DM increases the risk of heart failure, mostly due to CAD, in some cases it is secondary to diabetic cardiomyopathy [22]. Insulin-dependent DM patients have more comorbidities than non-insulin dependent patients, as reported in this study. Although the presence of insulin treatment is indeed a marker for more advanced disease, its underlying biological mechanism has not been fully elucidated. It may be related to the impact of a procoagulant imbalance, chronic exposure to high glucose levels, and direct effects of hyperinsulinemia. Interestingly, endogenous hyperinsulinemia has been associated with increased long-term mortality following MI in patients without diabetes [23]. Further studies are required to examine whether insulin-dependent diabetic patients should be included in risk stratification algorithms for patients who undergo CABG, and also whether they require more intense cardiovascular protective therapies with the newly available anti-diabetic drugs.

### Limitations

A selection bias could have been introduced by the fact that, while primarily the ACSIS registry included patients admitted only to cardiology wards and intensive cardiac care units nationwide, in the main it did not include patients hospitalized in internal medicine wards. There was insufficient anatomical information regarding the complexity of CAD, the specific artery involved, and the surgical techniques performed. Therefore, it is difficult to draw conclusions regarding the association between specific interventions in native arteries or grafts and clinical outcomes. Information regarding patients treated with insulin analogs compared with human insulin was lacking in the ACSIS registry, and therefore we could not draw any conclusions regarding specific treatment that could improve cardiovascular morbidity in insulin-dependent DM patients.

## Conclusions and clinical implications

We have shown that the presence of DM among patients with ACS who are referred to CABG is a powerful risk factor for long-term mortality, especially if the diabetic treatment strategy includes insulin. Accordingly, the high-risk population of insulin-dependent DM may require specific and/or more intense cardiovascular protective therapies after CABG. Further studies are needed to examine whether novel interventions, such as GLP-1 analogs or SGLT2 inhibitors, could improve the long-term outcomes of these patients.

## Data Availability

Data collected from the ACSIS national registry.

## Abbreviations

DM: Diabetes mellitus
CAD: Coronary artery disease
ACS: Acute coronary syndrome
CABG: Coronary artery bypass grafting
ACSIS: The Acute Coronary Syndrome Israeli Survey
MACE: Major adverse cardiovascular events
MI: Myocardial infarction
HR: Hazard ratio
CI: Confidence interval
